# 

**Published:** 1893-04

**Authors:** 


ESTABLISHED 1855. SUBSCRIPTION, $2.00.

LIBRARY Indexed S. G. O.

### ATLANTA MEDICAL AND SURGICAL JOURNAL.

OLD SERIES, VOL. XXVI. }

NEW SERIES, VOL. X. }

ATLANTA, GA., APRIL, 1893. No. 2.

LUTHER B. GRANDY, M. D., MANAGING EDITOR.

M. B. HUTCHINS, M. D., BUSINESS MANAGER.

